# The Genome of Enterobacter hormaechei Strain MG02, a 2,4-Dichlorophenoxyacetic Acid-Degrading Bacterium Isolated from Brazilian Soil

**DOI:** 10.1128/mra.01104-21

**Published:** 2022-02-28

**Authors:** Barbara Alvarenga Peckle, Sheila da Silva, José Roberto de Assis Ribeiro, Selma Soares de Oliveira, João Lídio Silva Gonçalves Vianez-Júnior, Ida Carolina Neves Direito, Andrew Macrae

**Affiliations:** a Programa de Pós-Graduação em Biotecnologia Vegetal e Bioprocessos da Universidade Federal do Rio de Janeiro, Rio de Janeiro, Rio de Janeiro, Brazil; b Instituto de Microbiologia Paulo de Góes da Universidade Federal do Rio de Janeiro, Rio de Janeiro, Rio de Janeiro, Brazil; c Center for Technological Innovation, Evandro Chagas Institute/SVS/MS, Ananindeua, Pará, Brazil; d Laboratório de Biotecnologia Ambiental (LBA) do Centro Universitário Estadual da Zona Oeste (UEZO), Rio de Janeiro, Rio de Janeiro, Brazil; Loyola University Chicago

## Abstract

Enterobacter hormaechei strain MG02 was isolated from a mixed culture collected from soil with a history of pesticide application. This strain degrades 2,4-dichlorophenoxyacetic acid (2,4-D). Here, we report on its genome, which has 4,923,875 bp and 55.4% G+C content.

## ANNOUNCEMENT

Strains of the genus Enterobacter are Gram-negative bacilli, non-spore forming, and belong to the family *Enterobacteriaceae*; they are widely distributed in nature (1). Enterobacter hormaechei subsp. *xiangfangensis* was described as degrading 2,4-dichlorophenoxyacetic acid (2,4-D) (2). Enterobacter hormaechei strain MG02 was isolated from soil that receives herbicides from the Cruzeiro Farm, Castelândia, Goiás, Brazil (18°04′49″S, 50°10′59″WGr). For isolation, a dilution (10^−1^) of 10 g of the soil was performed with 0.1% NaCl (wt/vol), 0.1% sodium pyrophosphate (wt/vol), and 0.1% (vol/vol) Tween 20 after incubation at 30°C and centrifuged at 180 rpm for 20 min. Then, serial dilution (10^−2^ to 10^−7^) in 0.85% saline was performed. We plated 100 μL of each dilution on 2,4-D MEMB medium (3). The strain was maintained in a minimal mineral medium with 2,4-D as the sole carbon source. A single colony was grown on LB medium at 28°C and was used for DNA extraction using the Purelink genomic DNA minikit (Invitrogen) and was DNA quantified by Qubit fluorometer.

The genome was sequenced using the Illumina MiSeq platform (Functional Genomics Center, ESALQ/USP) using 600 ng/mL genomic DNA (gDNA) for construction of a paired-end sequencing library (2 × 250 bp) with Nextera XT DNA sample preparation kit (Illumina, San Diego, CA, USA). The FastQC v0.11.9 (4) and Trim Galore v0.6.6 (5) tools were used to remove adapters and check and control read quality with a Phred quality score cutoff of >30. Genome assembly was performed combining *de novo* and scaffold mapping assembly techniques. SPAdes v3.15.0 (6) was used for the *de novo* assembly, and its output was used for assembly with MeDuSa v1.6 (7) along with 30 Enterobacter hormaechei complete reference genomes from NCBI. QUAST v5.0.2 (8) and CheckM v1.4.0 (9) were used to confirm quality of the assembly. The Prokaryotic Genome Annotation Pipeline (PGAP) v5.3 from NCBI (10–12) was used to annotate the genome. 16S rRNA taxonomic classification was done using NCBI BLASTn v2.12.0 using the nr/nt database (13). Genome taxonomy was done using Average Nucleotide Identity (ANI) v3.8.3, Pairwise Tetra-correlation (TETRA) v3.8.3, and the Tetra Correlation Search (TCS) v3.8.3 from JSpeciesWS v3.8.3 (14).

The sequencing resulted in 2,744,980 raw reads. The genome assembly resulted in 5 scaffolds and a genome length of 4,923,875 bp with 117.7× coverage and 55.4% G+C content, with an *N*_50_ value of 4,581,889 bp, and the *L*_50_ had 1 scaffold. Genome completeness was 99.37% with 0.71% contamination. The PGAP annotation resulted in 4,738 genes, 4,641 coding DNA sequences (CDS), 13 rRNAs, and 79 tRNAs. The 16S rRNA NCBI BLASTn search resulted in 100% query coverage and 100% identity with the genome of Enterobacter hormaechei strain AKB48 (NCBI accession no. GCF_012975085). The ANI based on BLAST (ANIb) and TETRA analyses resulted in scores of 98.4 and 0.9994, respectively, for Enterobacter hormaechei FDAARGOS 69. The TCS resulted in a 0.99992 score for Enterobacter hormaechei INSali10 and a 0.99983 score for Enterobacter hormaechei subsp. *steigerwaltii* CIDEIMsCOL3. For type strain Enterobacter quasiroggenkampii WCHECL1060, the results were 0.99166 (TETRA) and 86.82 (ANIb). Based on all of the above, strain MG02 was identified as belonging to Enterobacter hormaechei species. Default parameters were used for all software tools.

### Data availability.

The genome sequence of Enterobacter hormaechei strain MG02.2A has been deposited in GenBank under accession no. JAIQVY000000000. Raw reads were deposited in SRA under accession no. SRR15858125, BioProject under accession no. PRJNA757421, and BioSample under accession no. SAMN20961019.
